# Epidemiology and outcomes of non-HACEK infective endocarditis in the southeast United States

**DOI:** 10.1371/journal.pone.0230199

**Published:** 2020-03-10

**Authors:** Michael P. Veve, Eric D. McCurry, Grace E. Cooksey, Mahmoud A. Shorman

**Affiliations:** 1 University of Tennessee Health Science Center, Knoxville, Tennessee, United States of America; 2 University of Tennessee Medical Center, Knoxville, Tennessee, United States of America; Vita Salute University of Milan, ITALY

## Abstract

**Objectives:**

Infective endocarditis (IE) with non-HACEK Gram-negative (GN) organisms is rare, but associated with poor outcomes. The purpose of this study was to quantify the microbiology, treatment strategies, and frequency of poor outcomes in patients with non-HACEK GN IE.

**Materials:**

Retrospective cohort of adults with definite non-HACEK GN IE from 1/11-1/19. The primary endpoint was poor patient outcome, defined as a composite of all-cause death or infection-related readmission within 90-days of index infection.

**Results:**

43 patients were included: 51% patients were men, and the median (IQR) age was 40 (31–50) years. Forty patients reported injection drug use. The most common organisms were *Pseudomonas aeruginosa* (68%) and *Serratia marcescens* (9%). Seventy-six percent of patients received definitive combination therapy; the most common antibiotics used in combination with a β-lactam were aminoglycosides (50%) and fluoroquinolones (34%). Three patients discontinued combination therapy due to toxicity. Twelve-month, all-cause mortality and readmission was 30% and 54%, respectively. In multivariable logistic regression, variables independently associated with composite poor outcome were receipt of fluoroquinolone-based IE combination therapy and septic shock.

**Conclusions:**

Long-term mortality and readmission rates were high. Patients who received fluoroquinolone-based IE combination therapy more frequently developed poor outcomes than those who did not.

## Introduction

Non-HACEK Gram-negative (GN) infective endocarditis (IE) is a relatively rare condition associated with significant morbidity and mortality [1, 2]. While Gram-positive bacteria are typically the predominant causative organisms associated with IE, the increasing prevalence of invasive infections due to non-HACEK (i.e., organisms other than *Haemophilus* spp., *Aggregatibacter actinomycetemcomitans*, *Cardiobacterium hominis*, *Eikenella corrodens*, or *Kingella* spp.) GN bacilli have raised attention due to their propensity to develop and spread resistance, high attributable mortality, and associations with increased health care expenditures [3]. While the microbiology and ideal antibiotic treatment in non-HACEK GN IE is relatively unknown, current IE guidelines recommend cardiac surgery and prolonged combination antibiotic therapy as a reasonable approach to treatment [4]. A better understanding of the non-HACEK GN IE population is paramount in determining interventions targeted to improving patient outcomes.

Literature describing non-HACEK GN IE is lacking, particularly in the United States where injection drug use-related IE is common [5]. People who inject drugs (PWID) are traditionally thought to be at higher risk for non-HACEK GN IE [6–8], and many subsequently do not receive cardiac valve surgery due to the risk of recidivism [9]. Additionally, the collateral damage and toxicities observed with long-term use of the fluoroquinolone and aminoglycoside antibiotic classes that are recommended for use as combination therapies are concerning, and data supporting the use of combination therapy over monotherapy are conflicting [1, 2].

The purpose of this study is to quantify non-HACEK GN IE microbiology, describe patient characteristics, treatment strategies, and assess the frequency of poor patient outcomes.

## Methods and materials

This was a retrospective cohort study performed at the University of Tennessee Medical Center (UTMC), a level III trauma center and academic hospital located in Knoxville, Tennessee; this study was approved by the UTMC institutional review board and requirements for informed consent were waived. Patients were included if they met the following criteria: i) age ≥ 18 years, ii) hospitalization from 1/2011 to 1/2019, iii) IE diagnosis per ICD9/10 codes (421.1; I33.0), iv) definite IE per the modified Duke criteria, and v) positive blood or heart-valve cultures for a non-HACEK Gram-negative organism. Patients with previous 60-day history of IE, or polymicrobial GN IE with a Gram-positive or fungal organism were excluded. Patients were grouped into those who developed poor outcomes and those who did not; risk factors for poor outcomes were identified. Individual subjects were only included once, and if a subject was eligible over multiple admissions, the first admission meeting the case definition was identified as the index admission or infection.

### Study Data

Patients were identified for screening using pharmacy clinical decision support software; all data were extracted from the electronic medical record and collected using a standardized electronic case report form via REDCap (Nashville, TN) and hosted on secure internal servers.

The following data were extracted from the patient’s electronic medical record: past medical history, previous history of IE, injection drug use history, sex, race, age, select comorbid conditions, pre-hospitalization residence, insurance status, previous hospital exposure within the last 180 days prior to index admission, severity of illness (i.e., Pitt bacteremia score, presence of septic shock), hospital length of stay and discharge disposition, and patient outcomes. Injection drug use was assessed through i) patient admittance or self-identification as an injection drug user within the past 30-days, as denoted in the electronic medical record, ii) admittance to a history of substance use with a positive urine drug screen for illicit substances on admission, and/or iii) IE determined to be related to injection drug use by an infectious diseases physician at the time of care.

The following infection and treatment characteristics were also collected: type of endocarditis (i.e., native-valve, prosthetic-valve), affected heart valve(s), presence and type of septic emboli, concomitant infections, receipt of valve surgery, empiric and targeted treatment, and use of combination therapy. Combination therapy was defined as the receipt of at least two concomitant antibiotics targeted towards the index GN pathogen associated with IE for ≥ 72 hours, and was further classified by type of combination therapy used: β-lactam, fluoroquinolone, aminoglycoside, or other antibiotics.

Microbiological data collected included date and method of culture collection, culture susceptibilities of select antibiotics, dates of positive blood or valve tissue cultures if applicable, and follow-up blood cultures where available. Identification and susceptibility of blood isolates were determined via conventional methods with the VITEK^®^2 microbial identification system (bioMérieux, Marcy-l’Étoile, France) and according to the standard of care. Data collected included bacterial genus and species name and susceptibility interpretations according to Clinical and Laboratory Standards Institute (CLSI) breakpoints [10]. Non-HACEK Gram-negative organisms included the following: *Acinetobacter* spp., *Escherichia coli*, *Klebsiella* spp., *Enterobacter* spp., *Citrobacter* spp., *Serratia* spp., *Morganella* spp., *Proteus* spp., *Providencia* spp., and *Pseudomonas* spp. Organisms were considered multidrug resistant if they displayed resistance to one or more classes of antibiotics in three or more antibiotic classes that are active against the isolated bacteria [11].

### Outcome measures

The primary outcome of interest was the frequency of poor outcomes observed in patients with non-HACEK GN IE, defined as a composite of all-cause death or infection-related readmission within 90-days of the index infection. Infection-related readmission was defined as rehospitalization secondary to clinical worsening (i.e., new fevers, malaise) thought secondary to IE while on antibiotic therapy, infection relapse (i.e., new positive blood cultures with index organism) while on antibiotic therapy, or infection recurrence after antibiotic therapy was completed. Secondary outcomes included infection microbiology, all-cause mortality and all-cause hospital readmission within 1, 6, and 12 months, time to patient outcomes, and IE treatment strategies (i.e., frequency of cardiac surgery, use of definitive monotherapy or combination therapy, antibiotic selection and duration).

### Statistical analyses

Descriptive statistics were also used to describe the patient population, treatment strategies, and outcomes. In bivariate analyses, categorical variables were compared by Pearson’s chi-square or Fisher’s exact test and continuous variables were compared by the student’s *t*-test or the Mann-Whitney U-test. Classification and regression tree (CART) analyses were used to identify breakpoints in continuous variables associated with poor outcomes. Stratified analyses were performed to assess the presence/absence of effect modification. Any dichotomized variable found to have an association with poor outcome (*P*<0.2) or that was deemed clinically relevant *a priori* was considered for inclusion into a multivariable logistic regression model. Variables were manually entered into the multivariable model using a backwards-stepwise approach to determine independent associations with poor outcomes while controlling for potential confounders. The multivariable model was examined for goodness of fit using the Hosmer-Lemeshow test. All statistical analyses were performed using SPSS Software for Windows v.23.0 (IBM Corp, Armonk, NY).

## Results

A total of 43 GN IE cases were identified; 22 (52%) patients were men, and the median (IQR) age was 40 (31–50) years. A description of patient characteristics is depicted in Table 1. Forty (93%) patients reported a history of or active injection drug use, and the most commonly reported substances were opioids (77%) and amphetamines (14%). The most commonly injected opioids were oxymorphone (40%), oxycodone (21%), morphine (9%), heroin (9%), and unknown/not-specified (21%). Twenty-nine (67%) patients had a previous history of IE from a separate encounter prior to hospitalization, and the median (IQR) time from previous IE episode was 185 (73–508) days. Eight (19%) patients had previously been infected or colonized with a GN organism within the past year prior to index hospitalization.

**Table 1:** Characteristics of patients with non-HACEK Gram-negative infective endocarditis.

Variable, *n* (%) or median (IQR)	*n* = 43
**Patient Characteristics**	
Age, years	40 (31–50)
Sex, male	22 (51%)
Race, Caucasian	42 (98%)
No insurance	10 (23%)
Recent hospital exposure, 180 days	21 (49%)
Recent antibiotic exposure, 180 days	17 (40%)
Indwelling device on admission	11 (26%)
History of or active injection drug use	40 (93%)
Previous infective endocarditis	29 (67%)
Previous Gram-negative organism colonization or infection	6 (14%)
**Infection Characteristics**	
Length of stay, days	18 (10–35)
Admitted to ICU	18 (42%)
Septic shock	17 (40%)
Non-native valve IE	13 (30%)
Right-sided IE	27 (63%)
Septic emboli	28 (65%)

Abbreviations: ICU, intensive care unit; IE, infective endocarditis

The majority of patients were admitted to the general practice unit (58%), and 26 (60%) received care in the intensive care unit (ICU) at some point during hospitalization. The median (IQR) hospital length of stay and ICU stay was 18 (10–35) days and 4 (2–7) days, respectively. From a severity of illness perspective, 19 (44%) patients were found to be septic shock, and the median (IQR) Pitt bacteremia score was 1 (0–3). The infectious diseases consultation service was involved in each patient case.

All patients had evidence of endocardial involvement from diagnostic imaging. Native-valve IE was more common than prosthetic valve IE (70% vs. 30%), and the most frequent IE types were right-sided (63%), left-sided (35%), both left and right-sided (2%). The tricuspid valve was the most commonly infected valve overall (61%). Two (1%) patients had cardiac implantable electronic device-related IE. Forty-four organisms were isolated from 43 patients, and the most commonly identified pathogens were 68% *Pseudomonas aeruginosa*, 20% *Serratia marcescens*, 5% *Enterobacter cloacae*, 2% *Klebsiella oxytoca*, and 2% *Acinetobacter baumannii*, Table 2. Three (7%) organisms were considered multi-drug resistant based on *a priori* criteria (11), which were all *P*. *aeruginosa*. All organisms were identified from blood, except for one patient that had *P*. *aeruginosa* isolated from surgical heart valve cultures but was not bacteremic at time of blood culture collection.

**Table 2:** Microbiological data from patients with non-HACEK Gram-negative endocarditis, *n* = 43 organisms.

Organisms	*n* (%)	Drug Resistant Organisms
*Pseudomonas aeruginosa*	30 (68%)	3/30 (10%)
*Serratia marcescens*	9 (20%)	0/9
*Enterobacter cloacae*	2 (5%)	0/2
*Klebsiella oxytoca*	1 (2%)	0/1
*Acinetobacter baumannii*	1 (2%)	0/1

Forty (93%) patients received empiric therapy that had *in vitro* activity against the identified pathogen(s); all empiric therapy used had activity against *P*. *aeruginosa*, and 99% received an empiric anti-pseudomonal β-lactam. Forty-two (95%) patients received definitive therapy; one patient died prior to receiving definitive therapy. The majority of patients (*n* = 41, 98%) received a β-lactam as definitive therapy, which was most commonly an anti-pseudomonal cephalosporin, carbapenem, or anti-pseudomonal penicillin or cephalosporin/β-lactamase inhibitor combination. Of the three patients with multi-drug resistant *P*. *aeruginosa*, one patient received definitive ceftolozane/tazobactam, one received combination therapy of meropenem and amikacin, and one received ciprofloxacin. The median (IQR) duration of total antibiotic therapy was 41 (21–46) days.

Thirty-two out of 42 (76%) patients received at least 72 hours of definitive combination therapy, and the most commonly used agents in combination with a β-lactam were 50% aminoglycoside, 34% fluoroquinolone, and 13% received both. One patient (3%) received aminoglycoside and fluoroquinolone combination therapy. Three (9%) patients stopped combination therapy while hospitalized due to nephrotoxicity; all three patients were receiving aminoglycoside-based combination therapy. The median (IQR) time to toxicity was 13 (7–13) days. One patient who developed nephrotoxicity with an aminoglycoside had therapy changed to a fluoroquinolone with a β-lactam; the other two patients who developed nephrotoxicity received a single antibiotic for the remainder of therapy. The median prescribed duration of combination therapy was 34 (16–44) days.

Ten (23%) patients received valve surgery for IE management; the median (IQR) time to surgery was 8 (4–14) days. Most patients who received IE valve surgery presented with septic shock (62%), had left-sided IE (54%), and were generally young with a median (IQR) age of 43 (29–49) years. Eight (80%) patients had a history of or actively injected illicit drugs, and 1 (10%) was infected with multidrug resistant *P*. *aeruginosa*.

**Fig 1:**
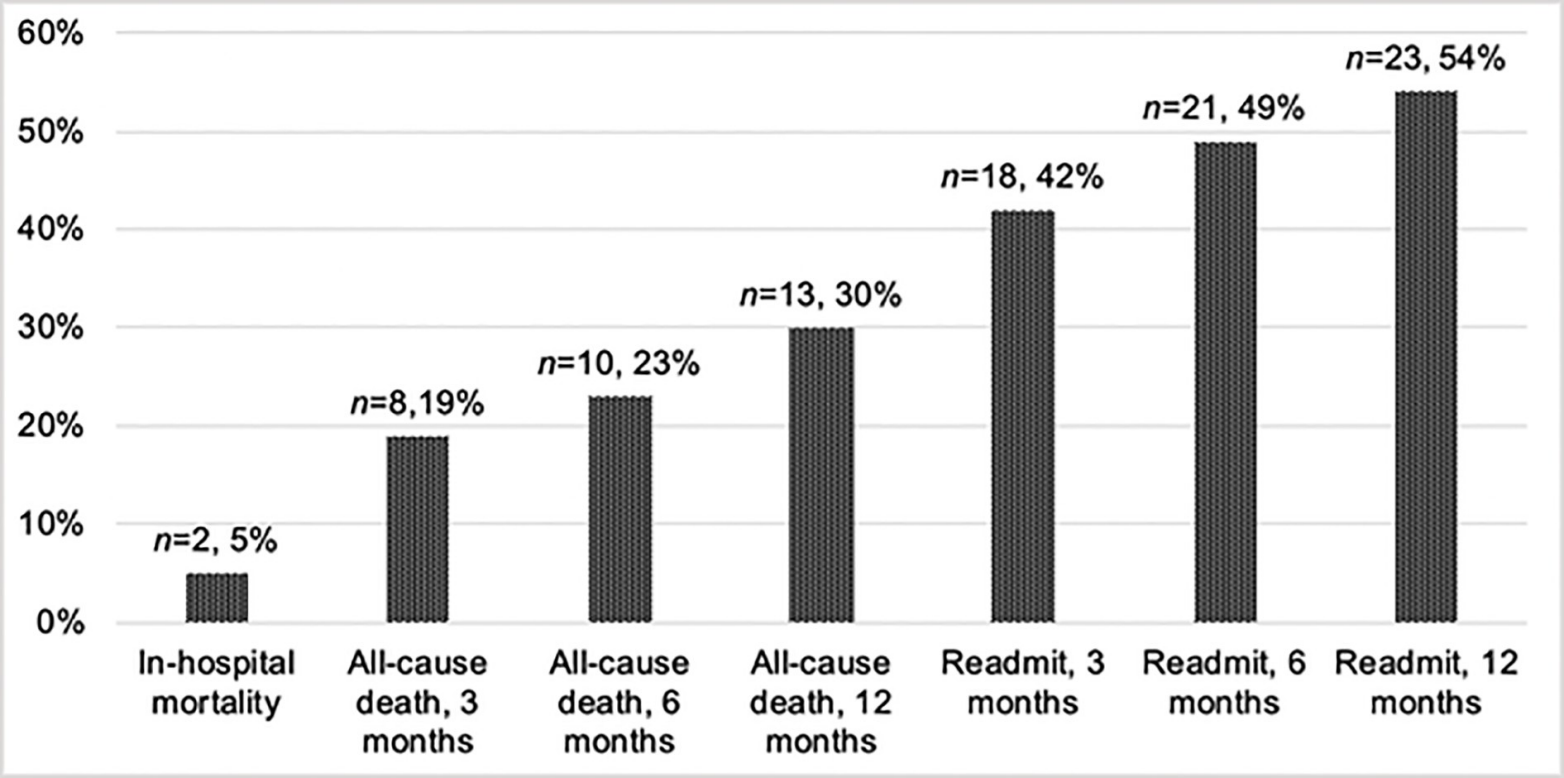
Outcomes of patients with non-HACEK Gram-negative infective endocarditis.

The primary composite outcome of all-cause death or infection-related readmission within 90-days from index infection occurred in 20 (47%) patients; 12 (60%) patients were readmitted secondary to the index infection, and 8 (40%) patients died. Of the 12 patients with a 90-day infection-related readmission, the majority of cases were due to infection recurrence (23%), followed by clinical worsening (7%), and infection relapse (5%). Two out of the 12 patients with a 90-day infection-related readmission had originally left the hospital against medical advice. The median (IQR) time to poor outcome after hospital discharge was 19 (10–63) days. Only two (5%) patients died while in the hospital, and twelve-month, all-cause mortality and readmission was 30% and 54%, respectively (Fig 1).

A sub-group analysis of the 10 patients who received valve replacement surgery showed that 6 (46%) suffered from a poor outcome within 90-days: 3 patients died, 1 patient had infection recurrence and died, and 2 patients had infection recurrence and survived. Additionally, 6 (46%) were readmitted due to any cause within 12 months, and 7 (54%) died within 12 months.

Results of the bivariate analyses and clinical rationale dictated the variables selected for inclusion into the multivariate regression model, Table 3. Other variables were excluded from the model because of unmet clinical or statistical criteria, to preserve the *n*:k ratio, or to prevent inclusion of variables that may covary. In the final parsimonious model, the receipt of fluoroquinolone-based IE combination therapy and septic shock were independently associated with an increase in poor patient outcome at 90-days post index infection.

**Table 3:** Variables associated with all-cause mortality or infection-related readmission within 90-days of index infection, *n* = 20.

Characteristic	Unadjusted OR (95% CI)	*P*-value	Adjusted OR (95% CI)	*P*-value
Receipt of IE combination therapy	14.6 (1.7–128.4)	0.004	Not Tested	--
Congestive heart failure	4.5 (0.8–25.6)	0.18	Not Tested	--
Receipt of fluoroquinolone-based IE combination therapy	4.2 (1.1–15.7)	0.03	6.7 (1.4–30.4)	0.02
Septic shock	3.4 (0.97–12.1)	0.05	5.2 (1.1–23.2)	0.03
*Pseudomonas aeruginosa* IE	2.6 (0.6–10.2)	0.17	Not Tested	--
Prior healthcare exposure, 180 days	0.35 (0.1–1.2)	0.13	Not Tested	--
Indwelling device on admission	0.33 (0.07–1.5)	0.18	Not Tested	--
IE surgery	0.4 (0.1–1.9)	0.31	Not Tested	--
Left-sided IE	0.4 (0.1–1.6)	0.21	Not Tested	--

Abbreviations: IE, infective endocarditis

Additional sub-analyses were performed to identify variables associated with all-cause 12-month mortality and readmission, Tables 4 and 5. In both of these analyses, receipt of fluoroquinolone-based IE combination therapy had some association with the outcome of interest, but was not found to be a predictor of all-cause, 12-month mortality.

**Table 4:** Variables associated with 12-month, all-cause readmission.

Characteristic	Unadjusted OR (95% CI)	*P*-value	Adjusted OR (95% CI)	*P*-value
*Pseudomonas aeruginosa* infection	13.1 (2.3–73.5)	0.002	13.9 (1.3–148.6)	0.03
Mental health disorder	3.2 (0.81–12.8)	0.11	Not tested	--
Injection drug use	1.3 (0.1–22.2)	1.0	Not tested	--
Mild liver disease	3.5 (0.95–13.2)	0.11	Not tested	--
Septic shock	0.35 (0.1–1.3)	0.13	0.2 (0.03–1.4)	0.11
Receipt of IE combination therapy	22.0 (2.4–199.9)	0.001	Not tested	--
Receipt of fluoroquinolone-based IE combination therapy	30.0 (3.3–269.4)	<0.001	33.9 (2.7–429.9)	0.006
Left sided IE	0.3 (0.1–1.4)	0.12	Not tested	--

Abbreviations: IE, infective endocarditis

**Table 5:** Variables associated with 12-month, all-cause mortality.

Characteristic	Unadjusted OR (95% CI)	Adjusted OR (95% CI)	*P*-value
Septic shock	6.7 (0.7–66.5)	Not Tested	--
Receipt of IE combination therapy	6.0 (0.7–52.9)	Not Tested	--
Congestive heart failure	5.6 (1.1–28.8)	2.9 (0.5–15.6)	0.24
Non-native valve IE	4.7 (1.1–19.1)	4.7 (1.1–19.1)	0.032
Previous IE	3.7 (0.7–19.6)	Not Tested	--
ICU on Admission	3.2 (0.8–12.4)	Not Tested	--
Left-sided IE	2.0 (0.52–7.6)	Not Tested	--
Receipt of valve surgery	1.7 (0.4–7.5)	Not Tested	--
*Pseudomonas aeruginosa* infection	0.96 (0.24–4.0)	Not Tested	--
Septic emboli	0.69 (0.14–3.5)	Not Tested	--
Mental health disorder	0.2 (0.05–1.3)	Not Tested	--

Abbreviations: IE, infective endocarditis; ICU, intensive care unit

## Discussion

This study found the rates of poor outcomes in patients with non-HACEK GN IE to be high. Multidrug resistant GN organisms were common, which may be related to previous health-care exposures and antibiotic consumption in a high-risk population of mostly PWID. Additionally, over half of the cohort were re-hospitalized, and roughly one-third of patients died after 12 months from index infection. These outcome data are consistent with previous reports of non-HACEK GN IE [1, 2,12]. In our cohort, patients who received definitive fluoroquinolone-based combination therapy and who presented with septic shock were significantly more likely to succumb to poor outcomes within 90-days post index infection compared to those who did not.

To our knowledge, this is the largest published cohort describing patient outcomes of non-HACEK GN IE from a single center, and even fewer reports have described outcomes in the United States population. Infective endocarditis from non-HACEK GN organisms has been primarily reported in PWID for many decades, with only a few reports of healthcare-associated cases [1, 2, 8, 12–15]. In our cohort, the majority of patients reported active or a history of injection drug use, which likely represents their main risk factor for IE. These patients were generally younger than previous published reports of healthcare associated non-HACEK GN IE, highlighting the devastating impact of this type of infection and its complications. In our cohort, other risk factors could include a history of previous IE, recent healthcare or antibiotic exposure, the presence of indwelling devices or prosthetic heart valve, and previous colonization or infection with GN organisms. These exposures have been identified in previously published reports, highlighting the complexity of this infection [1, 2, 16–19].

Non-HACEK GN organisms, except for *Salmonella* spp., have limited ability to form biofilms and have low affinity to endocardial epithelium, accounting for the limited number of reported endocarditis cases due to these pathogens. The presence of certain predisposing factors, including host factors, valve abnormalities, and presence of prosthetic material can facilitate the persistence of these pathogens within the vegetation [20, 21]. In our study, *P*. *aeruginosa* accounted for 68% of all cases, which can be explained by the high percentage of PWID in our cohort. Some reports suggest *P*. *aeruginosa* is a common contamination of the paraphernalia used for illicit drug injection with standing water, un-boiled tap water, or toilet water. In other reports, *P*. *aeruginosa* IE developed after instrumentation or the presence of intravascular catheters [16, 18, 19]. The second most common pathogen in our cohort was *S*. *marcescens*, accounting for 20% of all cases. Previous reports have associated this pathogen with injection drug abuse as well, and *S*. *marcescens* has the potential for inducible antibiotic resistance mechanisms that can make infection eradication challenging. This is further complicated by a lack of consensus regarding the most optimal treatment strategy [14, 22]. Interestingly, there were no patients with *Escherichia coli* endocarditis in our cohort, which was the commonest isolated pathogen in many previous published reports that can likely be attributed to the limited number of PWID in those reports or that most infections were due to other healthcare-associated exposures (i.e., device-related IE) [1, 2].

The majority of patients in our cohort received combination therapy with a β-lactam and aminoglycoside or fluoroquinolone, in accordance with previous published guidelines from the American Heart Association (AHA)/Infectious Diseases Society of America (IDSA) (4). There are limited data that describe outcomes of combination therapy compared to monotherapy for non-HACEK GN IE. A small observational study conducted by Morpeth et al. reported no statistically significant survival benefit among patients who received combination therapy [1]. Falcone and colleagues reported that survivors in their cohort of patients with non-HACEK GN IE were more frequently treated with a β-lactam, with or without an aminoglycoside, and found infection due to multidrug resistant organisms to be the only factor independently associated with in-hospital mortality [2]. In the present study, the receipt of combination therapy was associated with increased readmission rates, which is likely due to increased toxicities associated with non-β-lactam antibiotics. In particular, recent reports of significant adverse effects related to fluoroquinolone use, in addition to low-genetic barriers to resistance, may explain this finding [23]. These data, despite a limited number of patients and variability of organisms, suggest that a single appropriate antibiotic may be successful in treating non-HACEK GN IE.

A small proportion of patients in our cohort underwent early cardiac valve surgery. Cardiac valve surgery has been recommended in the AHA/IDSA guidelines as an integral part of the management of non-HACEK GN IE, especially with left-sided IE [4]. Other reports, however, including ours, have shown similar mortality rates between patients who did and did not receive surgery, emphasizing the role of appropriate antimicrobial therapy [1, 2]. The low rate of cardiac valve surgery may have also contributed to the shorter duration of hospitalization in comparison to other studies.

While the current study adds needed information related to the management and outcomes of non-HACEK GN IE, there are several limitations that should be acknowledged. The retrospective nature and small patient population of this study limit our ability to detect other variables or exposures associated with poor patient outcomes; however, the current study design remains a pragmatic approach given the nature of our research questions. A standardized electronic case report form was used to prevent information bias. Some patients with a previous history of IE may have had pre-existing valve vegetations that could lead to selection bias, but patients with a recent history (i.e., 60-day) of IE were excluded. As this study occurred in the United States, where injection drug use may be more common than other areas of the world, the results may have difficult generalizations to other countries. Composite endpoints can bias variables associated with poor outcomes, however this remains an efficient and pragmatic method to measure outcomes given limitations to sample size. Larger, multicenter studies focusing on this population are needed to validate our findings.

In conclusion, these results suggest that the morbidity and mortality of non-HACEK GN IE is significant. People who inject drugs are likely at high risk, and infected patients typically have prolonged hospitalizations, high readmission rates, and long-term survival is low. Patients who receive combination therapy may be at risk for higher rates of adverse reactions that can lead to poor outcomes. Developing strategies aimed at reducing the risk of this type of infection could decrease morbidity and the cost associated with these likely preventable infections [22].

## Supporting information

S1 Dataset (SAV)
